# Clustering of Giant Virus-DNA Based on Variations in Local Entropy

**DOI:** 10.3390/v6062259

**Published:** 2014-05-30

**Authors:** Ranjan Bose, Gerhard Thiel, Kay Hamacher

**Affiliations:** 1 Department of Electrical Engineering, IIT Delhi, Hauz Khas, New Delhi 110016, India; E-Mail: rbose.iitd@gmail.com; 2 Department of Biology, Technische Universität Darmstadt, 64287 Darmstadt, Germany; E-Mail: Thiel@bio.tu-darmstadt.de; 3 Department of Computer Science & Department of Physics, Technische Universität Darmstadt, 64287 Darmstadt, Germany

**Keywords:** information theory, genomic sequences, evolution, phylogeny, virus

## Abstract

We present a method for clustering genomic sequences based on variations in local entropy. We have analyzed the distributions of the block entropies of viruses and plant genomes. A distinct pattern for viruses and plant genomes is observed. These distributions, which describe the local entropic variability of the genomes, are used for clustering the genomes based on the Jensen-Shannon (JS) distances. The analysis of the JS distances between all genomes that infect the chlorella algae shows the host specificity of the viruses. We illustrate the efficacy of this entropy-based clustering technique by the segregation of plant and virus genomes into separate bins.

## 1. Introduction

The organization of genomes has evolved dynamically by a stochastic process comprised of mutation and selection. Another interesting open problem is to test whether the overall organization of genomes is subject to evolutionary pressures. In this paper, we examine and compare the local sequence entropies of several genomes. The traditional Shannon’s entropy is a measure for disorder, and is defined as 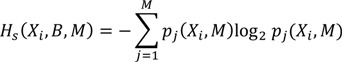, where, *X* is a random variable with realizations {*x*_1_, *x*_2_, …, *x_N_*} drawn from a discrete sampling space *S*. The *p*(*x_i_*) is the probability that *x_i_* occurs. In the case of genomes, *S* is the nucleotide alphabet of the DNA. When applied at the nucleotide level to genomic sequences, the sequence entropy for the full genome can be reduced to the question of CG-content [1,2]. This is a *global* property and related to the *mutation* rate, while the *selective advantage* reveals itself *locally* in, e.g., gene products such as regions coding for proteins.

The paper is organized as follows: Section 2 discusses the concept of superinformation, and lays the ground-work for entropy based clustering. In Section 3, we propose a method for clustering genomic sequences based on variations in local entropy. The conclusions are given in Section 4.

## 2. Superinformation Revisited

Shannon’s entropy represents the information content of the data in the *average* sense and some of the features, e.g., the local variations due to selection in particular, are lost. This shortcoming motivated Bose *et al.* [3] to introduce the concept of *superinformation*. The entire genomic sequence is subdivided into *N* blocks, of length *B* nucleotides each. Depending on the *local* characteristics of the data, the blocks have different information content (*i.e*., measure for randomness). The *i*^th^ block has entropy *H*(*X_i_*). By definition, *H*(*X_i_*) is a non-negative quantity. Then, a probability measure can be derived by the following algorithm:

(i) Construct the histogram of the *H*(*X_i_*) values, *i.e.*,

{*H_j_*(*X_i_*, *M*)} = Histogram ({*H*(*X_i_*)}), *i* = 1, 2, … *N* (1)

where, the Histogram function collects the elements of vector {*H*(*X_i_*)} into *M* equally spaced bins and returns the number of elements in each bin.

(ii) Form a probability measure by normalization:

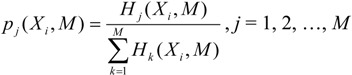
(2)


Then, *p_j_*(*X_i_*,*M*) is the frequency of *H*(*X_i_*) in the *j*^th^ bin.

*Superinformation* is then given by

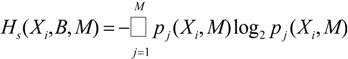
(3)
as a measure of the “entropy of entropy” and *B* defines the resolution at which this superinformation is calculated.

## 3. Clustering of Genomic Sequences

In this section, we propose a method for clustering genomic sequences based on variations in local entropy. The analysis includes several viruses from the family of *Phycodnaviridae* as well as eukaryotic organisms. The latter comprise higher and lower plants. The algae, which represent the lower plants, are specific hosts of the viruses [4,5]. For the relationship between the lower and the higher plants, it is worth noting that the Chlorella species belong to the green algae and are hence considered ancestors of the higher plants. The brown alga *Ectocarpus*, on the other hand, belongs to the *Heterocontophyta*, a group of algae, which separated very early from the green algae [6].

Viruses in the family of the *Phycodnaviridae* are huge, icosahedral viruses with large double-stranded DNA genomes. They replicate in a host specific manner in algae [4]. Virus EsV1 has, as a specific host, the brown alga *Ectocarpus siliculosus*. The viruses PBCV-1, NY2A and AR158 replicate exclusively in the chlorella species C. NC64A. The hosts for the remaining viruses are closely related Chlorella species for which we have no sequence information yet [4]. The viruses considered here are very suitable for this analysis. The annotation of the 330 kbp genome of the prototype virus *Paramecium bursaria* chlorella virus 1 (PBCV-1) identified *ca*. 366 protein-encoding genes and 11 *tRNA* genes. More than half of the predicted gene products resemble proteins from pro- and eukaryotes with a known function [4]. It is interesting to note is that these virus-encoded proteins are either the smallest or among the smallest proteins of their class; some are so much reduced that they represent not more than the minimal catalytic unit. A further interesting feature of these viruses is that they also have, unlike most other viruses, introns. Virus PBCV-1 for example has three types of introns: a self-splicing intron, a spliceosomal processed intron, and a small tRNA intron.

Accumulating evidence indicates that the chlorella viruses have a very long evolutionary history possibly dating back to the time when eukaryotes arose from prokaryotes [7,8,9]. They are predicted to have a common ancestor with the poxviruses (e.g., vaccinia virus), asfarvirues, iridoviruses, ascoviruses and mimiviruses [7,8]. Collectively, these viruses are referred to as nucleocytoplasmic large DNA viruses (NCLDVs). We now show that the local variations in entropy are not only very useful for clustering viruses and plant genomes, but may also suggest host specificity of viruses.

**Figure 1:**
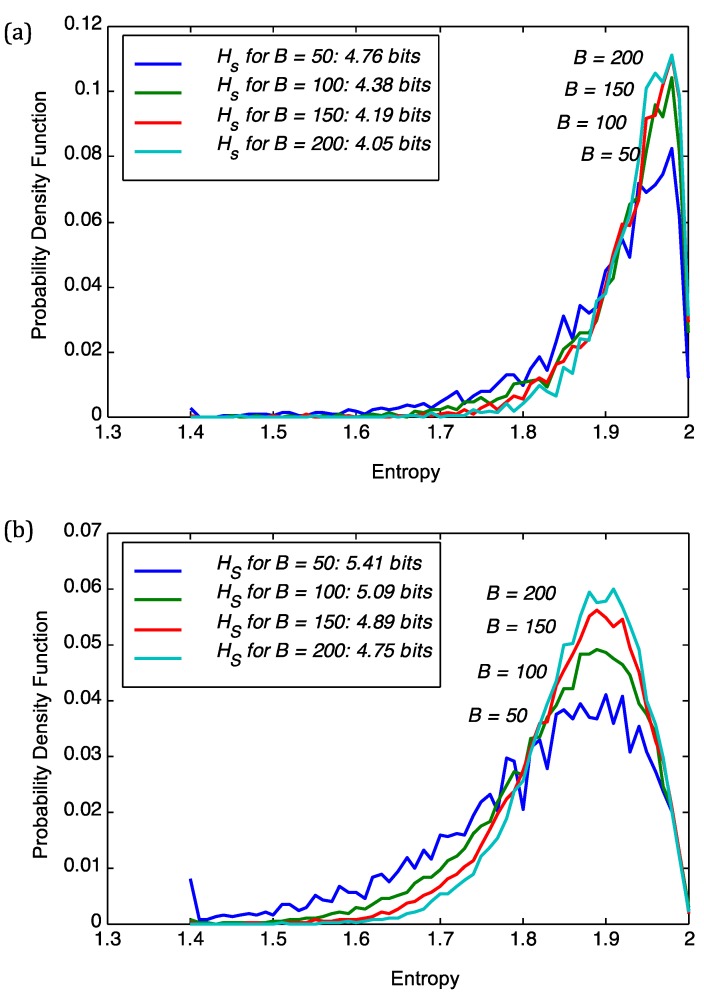
Sensitivity of the probability density function (pdf) of sequence entropies with respect to block size *B* for (**a**) the viral genome of PBCV1; and (**b**) *Chlorella* NC64A. We also show the superinformation *H_s_*.

We start with analyzing the superinformation content of viruses and plant genomes. The choice of block size *B* in Equation (3) was adjusted to stabilize the results for the superinformation, as shown in Figure 1. Intuitively, *B* defines the resolution at which superinformation is calculated. The figure shows the sensitivity of the probability density functions (pdfs) of sequence entropies with respect to block size *B* for the viral genome of PBCV1 and *Chlorella* NC64A. From Figure 1 we deduce that a choice of *B =* 100 implies stability of the subsequent analysis. For *B = 50* we obtained severe fluctuations in the pdfs as is the case for both PBCV1 and *Chlorella* NC64A in Figure 1. For *B = 100*, *B = 150*, and *B = 200* we obtain more regular histograms and therefore more stable superinformation values. However, the smaller the *B* the better the resolution and this we have opted to use *B = 100* in the subsequent parts as it provides stability and good resolution at the same time.

Similar sensitivities have been observed for the other seven plant viral genomes (AR158, ATCV, CVM1, FR483, MR325, NY2A, TN603), *Arabidopsis thaliana*, *Ectocarpus siliculosus* and EsV-1. Thus, we use this value of *B* in the subsequent parts of this study. It should be noted that the block-size (*B* = 100) is much smaller than the typical length of isochores (homogeneous domains) [10]. For example, in *Arabidopsis* genome, the length of GC isochore is of the order of 1 million base pairs [11].

In Table 1 we show the superinformation values for the genomic data of two plant hosts and their respective viruses. These results suggest the existence of a distinct pattern in these genomic sequences, implying differing selective pressures that shape the range of *local* variability (block entropies) and of *global* variability (superinformation). To derive evolutionary distances between all the sequences, the superinformation *H_s_* is, however, unsuitable. It should be noted that due to Chargaff's second parity G%~C%, A%~C%, plus the fact that GC% + AT% = 1, four base compositions (three are independent) in a block are reduced to one variable: GC%. So the entropy of a block, more or less has, a one-to-one correspondence with the GC%, and distribution of block-entropies are similar to distribution of a function of GC%. Thus the superinformation for the whole sequence corresponds to some measure of the window-GC% distribution (e.g., variance).

**Table 1:** Superinformation values *H_s_* for various genomic sequences. Note the tendency of increased *H_s_* for organisms in comparison to viruses.

Genomic Sequence	*H_s_* [Bit]
AR158	4.21
ATCV	3.62
CVM1	3.87
FR483	3.83
MT325	3.77
NY2A	4.22
PBCV1	4.38
TN603	3.65
EsV-1	3.54
*Arabidopsis thaliana*	5.27
*Chlorella* NC64A	5.10
*Oryza sativa*	4.74
*Ectocarpus siliculosus*	4.10

**Figure 2:**
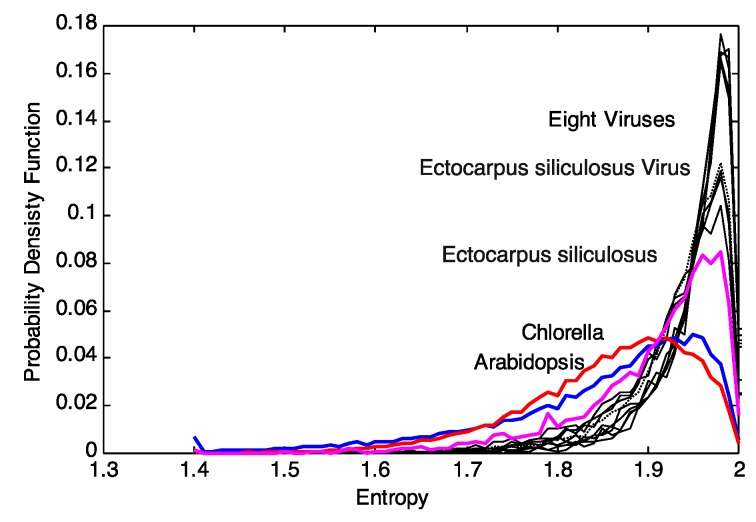
Plot of the probability density function (pdf) of the entropy for 8 plant viral genomes (AR158, ATCV, CVM1, FR483, MR325, NY2A, PBCV1, TN603), *Arabidopsis thaliana*, *Chlorella* NC64A, *Ectocarpus siliculosus* and EsV-1. The plots were obtained for a block size of *B* = 100.

Continuing with the rationale above that the distribution of *local* sequence entropies is a signal for evolutionary pressure of the environment, we decided to take one step back and use these distributions of block entropies *p_j_*(*X_i_*,*M*) instead. These distributions describe, in detail, the *local* variability of the sequences and thus any distance of such *p_j_*(*X_i_*,*M*) clusters entities based on the variability of entropies. The distributions of the block entropies of the genomic sequences are shown in Figure 2. Upon visual inspection, there appears to be a distinct pattern for viruses and plant genomes. The distributions for plant genomes show a larger variance as compared to that of the viruses. This motivates us to explore clustering based on the distributions of block entropies. We note that characterization of sequences based on the distribution of their sub-sequences have been explored earlier [12,13,14].

We can quantify the differences in the respective *p_j_*(*X_i_*, *M*) by generalized Kullback-Leibler divergences [15], the Jensen-Shannon-distances in particular [16] as discussed in [17,18]. The Jensen-Shannon-divergence, *D_JS_*(*p*,*q*), between the entropy distributions *p_i_*(*X_i_*, *M*) and *q_j_*(*X_i_*, *M*) for two data sources is given by

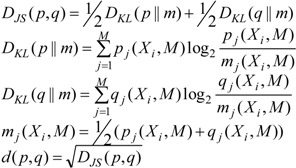
(4)
Here, *m_j_*(*X_i_*, *M*) is an “intermediate” distribution and the *D_KL_*(*p*||*m*) and *D_KL_*(*q*||*m*) are the Kullback-Leibler distances between *p* and *m* or between *q* and *m*, respectively. A suitable metric for clustering is then *d* (*p,q*) [16]. The application of Jensen-Shannon distance to DNA has also been carried out by previous researchers [19].

**Figure 3:**
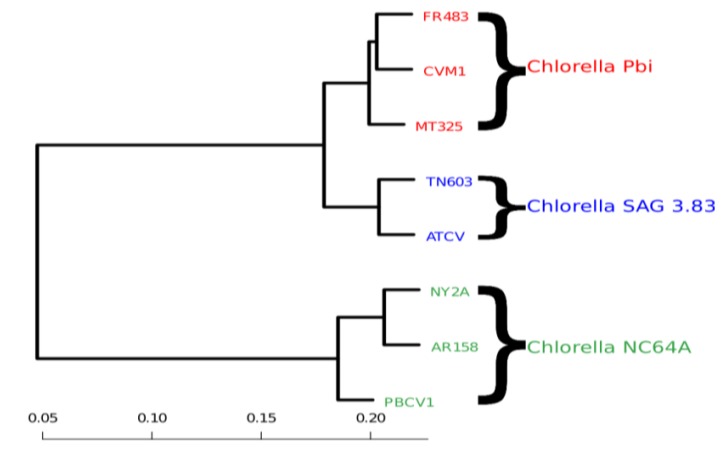
Clustering of viral genomes, annotated by their respective hosts—in this case variants of the *Chlorella* algae. The branch lengths are proportional to the phylogenetic Jensen-Shannon distance of Equation (4), the scale is indicated at the bottom.

We first computed the distances *d*(*p,q*) between all genomes that infest chlorella algae. We then used distance-matrix based clustering [20] as implemented in the statistical software R (R2009). In Figure 3, we clearly see that the genomic variability is able to reflect the host specificity of the viruses. The viruses that have a common host are closer to one another in terms of their block-entropy distributions. For example, the viruses FR483, CVM1 and MT325 that specifically infect Chlorella Pbi, are clustered together. The viruses PBCV-1, NY2A and AR158 that replicate exclusively in the chlorella species C. NC64A, are clustered together. This is a valuable finding and probably suggests that the respective host environment gives rise to unique selective pressures. It should be noted that (i) MT325 and FR483 are strongly related genomes sharing 94% of their genes with an average 86% amino-acid sequence identity and an almost identical gene order [21] and that (ii) both of these genomes are related to PBCV-1, but to a lesser extent (82%, 50%, weak gene order conservation). Hence, the identification of MT325 and FR483 (infecting the same host) to be more closely related than they are to PBCV-1 would be captured by sequence alignment tools.

**Figure 4:**
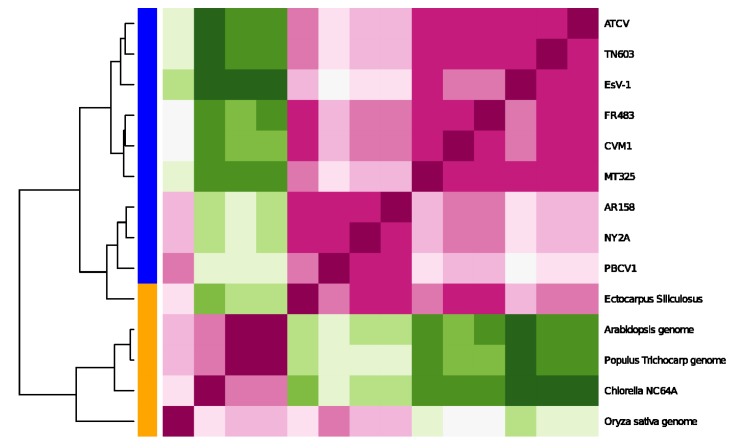
Clustering by the entropy-variability distance of Equation (1) for the extended genomic sequence set. We show the distance matrix *d*(*p,q*) for any genomic sequence pairing (*p,q*) for each genomic sequence with respect to each sequence (center plane, red = small values, green = high values). The bar on the left indicates whether the sequence is of viral origin (blue) or a living organism (orange). Note that the tree is not rooted.

These findings motivated us to augment the data set by including: (a) the host genome itself (*Chlorella* NC64A, [22]); (b) an independent host-virus genome pair (*Ectocarpus siliculosus*, [5]); and (c) other plant genomes, of which only a few are available up to this day. The plant genomes that we have included in our study belong to Populus trichocarpa (black cottonwood), *Oryza sativa* (Asian rice) and *Arabidopsis* (a small flowering plant). This set-up allows us to investigate whether the evolution of the host genomes is subject to the same evolutionary dynamics, leading to similar variabilities in the *local* entropies, as is the case for their respective viruses. By this, we can judge whether there exists differential evolutionary pressure on host and pathogenic genomes. Figure 4 shows the results of this experiment.

We see a clear separation of the genomic sequences when we cluster based on local entropy of the genomic data. In particular, there is a clear separation between viral and host genomes. This is a very interesting result and shows that clustering based on local entropy is able to assign the plants and viruses to different groups. The green alga and the higher plants form a sub-clade, while the plant viruses form a separate one. This comes out very clearly in Figure 4. The other interesting observation is that the brown alga (*Ectocarpus siliculosus*) is separate from the green alga (*Chlorella*) and other higher plants (Populus trichocarpa, *Oryza sativa* and *Arabidopsis*). This is consistent with the fact that these plants separated very early in evolution [6]. The fact that the genome of *Ectocarpus silicolosus* includes the entire genome of the respective virus EsV1 [5] may contribute to this separate position. Interestingly, the viruses infecting *Chlorella* subspecies include the *Ectocarpus silicolosus* virus; this occurs even though the viruses have very different lifestyles [4].

## 4. Conclusions

In this paper, we have proposed a novel method for clustering genomic sequences based on variations in local entropy. The clustering of the genomes on the basis of the Jensen-Shannon distances clearly brings out the host specificity of the viruses, *i.e.*, the viruses that have a common host are closer to one another in terms of their block-entropy distributions. The proposed entropy-based technique is also able to segregate plant and virus genomes into separate bins. Our clustering technique also resulted in brown alga being separate from the green alga and other higher plants, which is consistent with the fact that these plants separated very early in the process of evolution.
